# Enzyme-displaying spores as a novel strategy for mixed fiber textile recycling

**DOI:** 10.3389/fsysb.2025.1603731

**Published:** 2025-05-30

**Authors:** Matti Lehmann, Max Herrmann

**Affiliations:** Faculty of Mechanical Science and Engineering, Institute of Natural Materials Technology, Technical University Dresden, Dresden, Germany

**Keywords:** *Bacillus* *subitilis*, textile recycling, blended textiles, bioprocess engineering, spore surface display

## Abstract

Global textile manufacturing practices are responsible for an increasing amount of textile waste that pollutes our planet. Mixed fiber blends pose a recycling challenge due to their heterogeneous structure. Current mechanical, chemical, thermochemical and enzymatic strategies suffer from several limitations such as high energy costs, extensive pre-treatment requirements and enzyme instability. This mini-review aims to present recent developments in the research field and to introduce Spore Surface Display (SSD) technology as a new biological approach for mixed textile degradation. SSD allows enzymes to be anchored on the robust bacterial spore surface, immobilizing multiple enzymes required for simultaneous cotton-polyester degradation into their respective monomers. The mini-review also includes an initial proposal for a process design suitable for a full mixed textile degradation process using this synthetic biology approach.

## Graphical Abstract

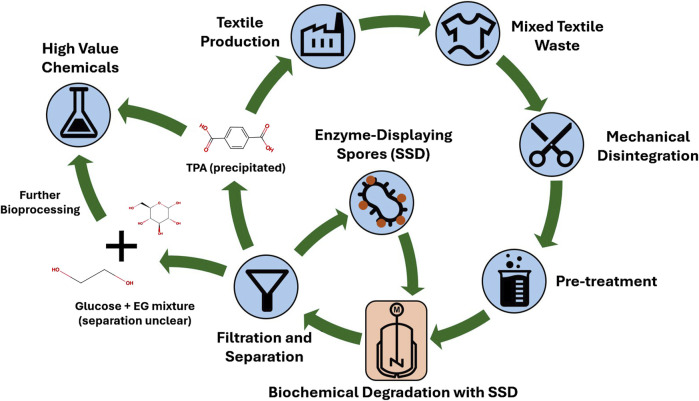

## 1 Introduction to textile waste and mixed fiber recycling

In recent years, the textile industry has shown rapid growth globally. The fast fashion business model is based on selling fashionable clothes at low prices (Caro and Martìnez-De-Albèniz, 2015). It comes with rapid production cycles, which lead to an increase in clothing waste. In 2020, global clothing production reached 109 million tonnes, and it is projected to rise to 145 million tonnes by 2030 (European Parliament, 2024). Every year, approximately 92 million tons of textile waste are produced globally (Ruiz and Arabella, 2024), of which 87% are incinerated or landfilled, contributing to approximately 10% of global carbon emissions (Fan et al., 2024).Only 1% of this textile waste is recycled into new garments (European Parliament, 2024). Recycling blended textile waste poses further technical challenges compared to single-material textiles. Most blended textiles consist of polyester and cotton fibers, which can be structurally regarded as polyethylene terephthalate (PET) and cellulose, respectively (Egan and Salmon, 2022). Recycling mixed textiles, such as cotton-polyester blends, remains challenging and typically requires the separation of each composite fiber type (Loo et al., 2023). Hence, the development of new recycling approaches is necessary.This mini-review provides a compact introduction to current strategies in mixed textile recycling, particularly for cotton-polyester blends. In addition, recent advances in Spore Surface Display (SSD) technology are discussed, which is then applied to the issue of textile recycling through a process engineering perspective.

## 2 Strategies for mixed fiber textile recycling

Mixed fiber textiles consist of at least two different polymers, such as cotton, polyester, wool, or nylon. The respective polymers are intertwined during fabric formation, resulting in a more durable product but also presenting recycling challenges. The heterogeneous microstructure hinders conventional separation approaches (Harlin et al., 2024).

Consequently, mechanical recycling, the dominant approach for recycling single fiber textile waste, is not suitable for mixed fibers (Chen et al., 2023). In essence, mechanical disintegration is a downcycling approach, making it suitable only for limited use cases such as insulation (Hussain et al., 2023). To recover high-value monomers in the fiber blends and return them into production cycles, chemical, thermochemical, or enzymatic approaches have attracted increasing interest.Among mixed fibers, textile recycling has seen the greatest progress for the dominant species of cotton-polyester blends. Current efforts in cotton-polyester blends focus on the separation of the synthetic compound from the natural fibers by degrading cellulose, thereby enabling retrieval of the higher-value synthetic compound.

Recent advances have significantly improved the deconstruction of blended textile waste through chemical or enzymatic means. An Overview is given in Table 1. Chemical recycling techniques have been shown to selectively remove cotton or PET from cotton-polyester blends. For instance, superconcentrated hydrochloric acid successfully depolymerised PET into Bis(2-Hydroxyethyl) terephthalate (BHET), making removal of the cotton fraction possible (Andini et al., 2024). Less harsh sustainable solvents—including 1,5-diazabicyclo [4.3.0] non-5-enium acetate ([DBNH] [OAc]) ionic liquids—have shown promising dissolving effects on the cellulose fraction (Haslinger et al., 2019). Betaine-based Deep Eutectic Solvents were used to catalyze cotton-polyester blend recycling, selectively targeting polyester and preserving the integrity of cotton fibers (Liu et al., 2022). Demonstrated that acid- and base-free depolymerization of PET with ethanol is possible, when catalyzed with ${\text{FeCl}}_{3}$ or ${\text{FeBr}}_{3}$ (Nor Wahida Binti Awang et al., 2025).

**TABLE 1:** Overview of mixed textile recycling approaches.

Approach	Outcomes	Notes	References
[DBNH] [OAc] Ionic Liquid	Selective cotton targeting, polyester preserved	More sustainable, less harsh solvent	Haslinger et al. (2019)
Betaine-based Deep Eutectic Solvents	Selective polyester targeting, cotton preserved	High yield, mild conditions, environmentally friendly	Liu et al. (2022)
Solid-State Mechanoenzymatic	Selective polyester targeting, cotton preserved	Enzyme stability challenges due to physical agitation	Egan et al. (2023)
Superconcentrated HCl Depolymerization	Selective polyester targeting, cotton preserved	Uses harsh chemicals	Andini et al. (2024)
Hydrothermal Treatment	Selective cotton targeting, polyester preserved	Requires high temperatures, making scale-up costly	Matsumura et al. (2024)
Gamma-Valerolactone Co-hydrolysis	Sequential hydrolysis and glycolysis, 75% glucose yield, 78% PET conversion	Allows lower acid concentrations	Zhang et al. (2024)
Ethanolamine depolymerization ( ${\text{FeCl}}_{3}$ and ${\text{FeBr}}_{3}$ )	Selective polyester targeting, cotton preserved	Mild conditions, good selectivity	Nor Wahida Binti Awang et al. (2025)

Another recycling approach, called hydrothermal processing, selectively degrades cotton components at high temperatures (220°C–230°C) while maintaining fabric shape (Matsumura et al., 2024). Meanwhile, solid-state mechanoenzymatic processes combine physical agitation with engineered enzymes. This approach promotes selective depolymerization of PET in mixed textiles, leaving the cotton fraction largely intact (Egan et al., 2023). Unlike previous examples, Gamma-Valerolactone solvent systems were reported to successfully co-hydrolyze cotton-polyester blends at lower acid concentrations—rather than focusing on the degradation of one fraction and separating the other (Zhang et al., 2024).

While these methods have demonstrated effectiveness in breaking down complex blends, key limitations remain. The need for extensive pre-treatment requirements (e.g., milling or chemical oxidation) and the inherently slow kinetics due to the intricate synthetic fibre structure significantly limit process efficiency. Additionally, for enzyme-based approaches, enzyme deactivation and product inhibition further complicate the recycling of heterogeneous materials (Osbon and Kumar, 2019). Enzyme deactivation primarily results from two factors: first, utilizing enzymes beyond their normal operating conditions (at extreme pH-levels or temperatures) detrimentally affects enzyme activity; and second, product accumulation inhibits enzyme activity, reducing overall process efficiency (Jönsson et al., 2021). Immobilization techniques are currently being explored to enhance enzyme stability and recyclability (Osbon and Kumar, 2019).

## 3 The case for spore display technology

Some microorganisms can temporarily turn into spores when they witness harsh conditions. Their resistance to environmental stress (chemicals, heat, ultraviolet radiation) is mainly achieved through the spore coat (Driks, 2016). It serves as a defensive barrier, protecting the spore from toxic substances and predatory microbes (Balassa, 1971; Klobutcher et al., 2006). Meanwhile, the envelope is also permeable for certain substances like nutrients that pass through to receptors in the inner membrane. This can trigger a signal for germination, making the spore turn back into a normal cell (Gao et al., 2023). The spore coat has a very complex structure, containing at least 80 proteins arranged in four layers: basement layer, inner coat, outer coat, and crust (Driks, 1999).

The SSD technology is an innovative approach, taking advantage of the inherent characteristics of such endospores. During this technology, a protein of interest is joined together with the spore surface. The goal of anchoring proteins in the spore coat is to improve their stability and functionality (Zhang, 2019). The protein benefits from the spores’ ability to survive harsh environments while maintaining their resistant properties after genetic modification (Hinc, 2013). Through these strategies, proteins may be protected from heat-induced denaturation and organic solvents. Meanwhile, the spores’ characteristics allow for easy recovery and reusability, something very difficult to achieve with live cell cultures. Therefore, SSD technology represents a sustainable and cost-effective tool in biotechnology (Bartels, 2018; Sheldon, 2007).

Of all sporulating microorganisms, *B. subtilis* has been at the forefront of SSD (Cutting, 2011; Westers et al., 2004; Isticato et al., 2001). *B. subtilis* is capable of forming highly resistant endospores while offering a very efficient secretion capacity (Cui et al., 2018; Errington, 1993; Harwood, 1992; Hecker et al., 1996). Thus, *B. subtilis* offers essential features necessary for a suitable host organism for the expression of heterologous proteins. When entering the sporulation phase, target proteins are synthesized along with spore-coat proteins that serve as anchors. Fusing the target protein with a sporulation-dependent promoter such as *PcotYZ* of *B. subtilis*, expression is guaranteed under sporulation. This anchor *PcotY* is located in the crust, the outermost layer of the spores. *PcotY* is well suited for protein immobilization and provides the highest activity for SSD (Bahrulolum and Ahmadian, 2018; McKenney et al., 2013; Lin et al., 2020).

### 3.1 Limitations and challenges

Despite its promise, SSD technology faces challenges, such as ensuring the genetic stability of the modified spores, overcoming regulatory hurdles, achieving adequate enzyme loading and scaling up the process for industrial applications (Lin et al., 2020).

## 4 Reasoning for mixed fiber processing

Current strategies for mixed fiber textile recycling separate cotton-polyester blends, i.e., no simultaneous recycling of both polymers. Thus, the degradation process is either conducted under optimal conditions for cellulose or PET leaving products, by-products and the not degraded polymer behind depending on the actual process. However, SSD technology can introduce several catalysts, namely, enzymes, to the process at the same time, paving a new way to transform both polymers to their monomers within one step. As enzymatic approaches to clothing recycling have seen increasing interest, recently, due to their ability to work under lower temperatures, reducing the risk and energy cost of conventional thermochemical approaches (Jönsson et al., 2021), enzymes themselves also come with a production cost. Their efficiency is highly temperature-dependent, and they are typically single-use in their free form (Madhu et al., 2017; Andreaus et al., 1999). In contrast, SSD technology can be used multiple times as long as the spores have not germinated yet, is resistant to harsh environments as found in industrial contexts and inherently produces the necessary enzymes itself.

The enzymes of interest for mixed fiber degradation are PETases (polyethylene terephthalate hydrolases, EC 3.1.1.101) catalyzing the formation of terephthalic acid (TPA) and ethylene glycol (EG) from PET and three types of cellulases ( β -glucosidases, exo- and endoglucanases) catalyzing the degradation of cellulose to glucose (Egan and Salmon, 2022; Behera et al., 2017). Endoglucanases (endo-1,4- β -D-glucanases, EC 3.2.1.4) cleave internal β -1,4-glycosidic bonds in cellulose, thereby releasing reducing and non-reducing chain ends. Exoglucanases, also called 1,4- β -cellobiosidase, remove cellobiose, a disaccharide, from the exposed ends in the crystalline region. Some exoglucanases work only on reducing ends (EC 3.2.1.176) while others cleave cellobiose only from non-reducing ends (EC 3.2.1.91). Finally, β -glucosidases (1,4- β -glucosidases, EC 3.2.1.21) hydrolyze cellobiose or cello-oligosaccharides to glucose monomers. As there exist a fungal and a bacterial version of the aforementioned cellulases, only the bacterial versions are recommended to use for SSD regarding the bacterial expression system of *B. subtilis*. Thus, the enzyme expression during sporulation is more likely and, furthermore, it is even a producer of endoglucanase itself (Aa et al., 1994). Finally mounted on the spore surface, the PETase performs its degradation procedure in a two-step manner, i.e., an acylation reaction followed by a deacylation reaction (Burgin et al., 2024). The three types of cellulases, however, work synergistically where endoglucanase creates randomly internal free chain ends that are processively degraded by exoglucanase to cellobiose which is then cleaved into two glucose molecules by β -glucosidase. As cellobiose inhibits the activity of endo- and exoglucanase, especially the last step has a great effect on the cellulose degradation rate (Wu et al., 2018).

In contrast to other biocatalyst systems that do not use the enzymes’ free form, the application of SSD technology comes with several advantages. The most significant difference with established immobilization platforms is that the connection between protein and spore crust is inherent because it is created during the spore formation, whereas for conventional immobilization methods the enzyme and the enzyme carrier must be produced separately. Therefore, no production of toxic immobilization materials as carbon tubes or metal powders is necessary (Khafaga et al., 2024). In addition, spores are still small in size (1.2 µm long and 0.8 µm wide) compared to other immobilization platforms (Chada et al., 2003). This makes it more likely for displayed enzymes to be able to access dense textile fibers, which is crucial for efficient textile degradation. Moreover, the spores’ protection against harsh environmental conditions makes them suitable for industrial applications (Lin et al., 2020). Additionally, enzyme purification is not necessary, as the enzymes are directly displayed on the spore surface. Finally, displaying the enzymes might also increase the stability of proteins (Lin et al., 2020; Zhang et al., 2019).

## 5 Bioprocess engineering realization

**FIGURE 1:**
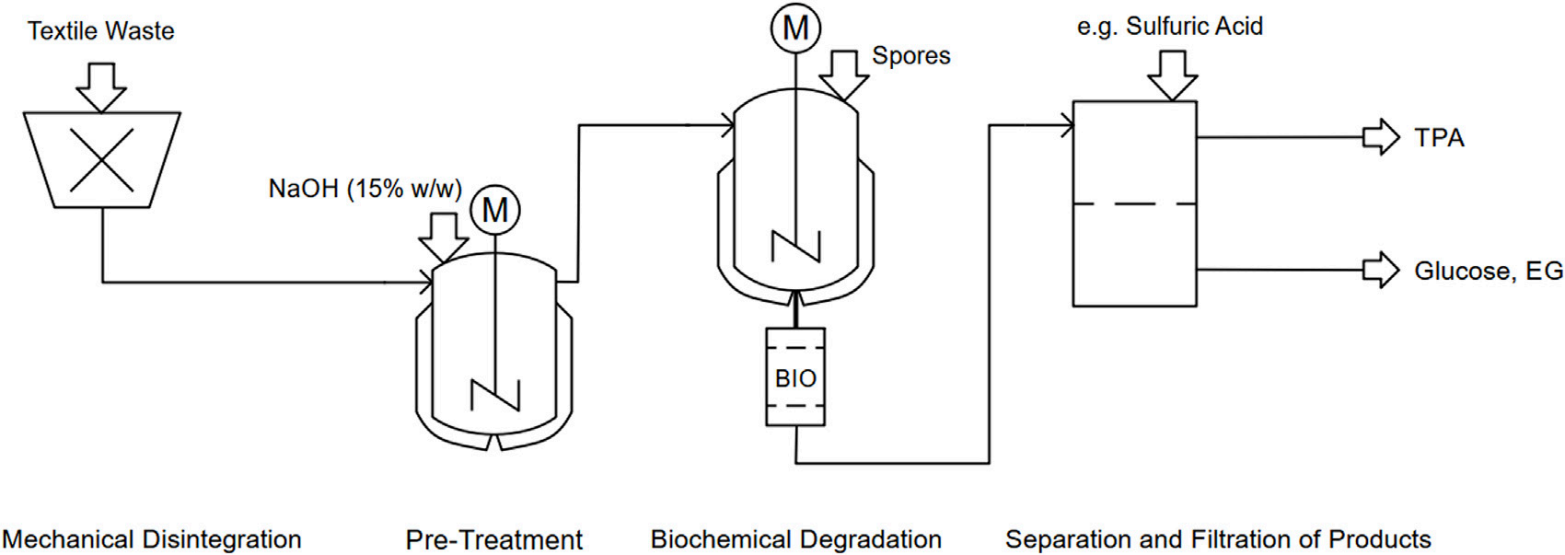
Piping and Instrumentation Diagram for the proposed mixed textile recycling approach.

Despite the findings on the SSD’s suitability, it remains challenging to implement it in an appropriate process. Inspired by existing recycling technologies, the process could be divided into four main steps: mechanical disintegration, pre-treatment, biochemical degradation and separation/filtration of the products (see Figure 1).

### 5.1 Mechanical disintegration

Textile waste comes in various forms, from industrial scraps to unsold clothing. To guarantee high-quality products and a stable process, homogeneity of the substrate is desired to ensure equal amenability to the displayed enzymes. Delivered substrates must be sorted to manually remove insoluble parts such as buttons, zippers and other non-textile components. It must be mechanically disintegrated to destroy the fiber structure, which is best done with a shredder and hammer mill. Producers of such potential feedstock are companies such as ALTEX and Soex, which sell it mainly as a filler to industrial customers for other applications.

### 5.2 Pre-treatment

Mechanical disintegration leads to macroscopic changes in the fiber structure but not microscopic changes (Cao et al., 2015). While this is not as important for PET, it is critical for cellulose. To further increase the amenability of the enzymes, a treatment is required that breaks the crystalline structure of the cellulose. Although the substrate composition is slightly different, there are several methods to achieve this (Chen et al., 2019; Ang et al., 2012; Arlai et al., 2025). Some of these methods require either special chemicals or mills, making these approaches not suitable for industry. However, one treatment stands out due to its high enzymatic hydrolysis yield and relative ease of implementation. It consists of using a 15% NaOH solution at 120°C in an autoclave (Boondaeng et al., 1964).

The shredded textiles absorb the lye, which deprotonates the hydroxide groups of the cellulose, increasing its overall charge. This weakens the intermolecular forces within the cellulose, as equally charged particles repel each other. Compared to other common methods, the PET and cellulose fibers do not react too strongly to the alkaline treatment, so the chemical structure is largely retained (Yokota et al., 2022). Another pre-treatment - normally applied to wood - is the combination of chemical and physical forces, which come together in the steam explosion process. The cellulose structure is disrupted more efficiently, resulting in an even more damaged polymer (Auxenfans et al., 2017). For potential implementation, a comparison of the degree of efficiency between an autoclave and a steam explosion machine is necessary to identify the better strategy.

### 5.3 Cultivation of spores

Before the actual depolymerization of cellulose and PET, the biocatalyst, the spores, must be cultivated. The production of enzyme-displaying spores requires careful cultivation strategies, as the *B. subtilis* strain used is genetically modified and therefore competes with its potentially plasmid-free offspring. As with most whole-cell catalysts, the seed train starts with pre-cultures of increasing volume, followed by a main culture optimized for biomass production. A fed-batch reactor is ideal for this purpose, with optimal growth of *B. subtilis* occurring aerobically at 37°C at pH 7.0 with glucose as the primary substrate (Luo et al., 2010). Once sufficient biomass has been produced, the sporulation process is initiated by transferring the culture to conditions that promote spore formation, such as depletion of carbon sources. The resulting spores can be harvested, dried if necessary, and stored until needed for the textile degradation process.

### 5.4 Biochemical degradation

The conversion of cellulose into glucose and PET into TPA and, EG is the main focus of the process. Since the pretreatment solution is highly alkaline, the pH must be adjusted to the optimum for the enzymes on the spore crust. In addition, the current enzymatic degradation rates of PET are still not economically viable (Chen et al., 2022). As any chemical reaction, the degradation of PET is also temperature dependent. It is characteristic of PET that high reaction rates are observed around 70°C. This temperature - also known as the glass transition temperature - reduces the density of PET and thus improves the accessibility of enzymes to the PET surface (Akram et al., 2024). Only some modified PETases can operate in such an environment, but their industrial application has not yet been demonstrated (Stevensen et al., 2025). Due to this uncertainty in affinity and activity, the robust nature of spores inspired their choice as an anchor for the enzymes in the first place.

### 5.5 Separation and filtration of products

As the spores are only the catalyst and not part of the desired product, they must be removed. This can be done using conventional filtration technologies such as tangential flow filtration. It is efficient for processing large volumes of liquid and operates at lower pressures than other filtration methods. This makes it ideal for separating spores from the rest of the solution on an industrial scale. The lower pressure even helps to preserve the filtration membrane, so the time between failures is relatively longer. Nevertheless, these advantages are accompanied by a higher energy demand and operational complexity compared to other filtration technologies.

After spore separation, managing the separation of the three-component mixture - glucose, TPA and, EG - is the next challenge. TPA can be precipitated under acidic conditions, while such conditions do not affect the solubility of glucose or, EG (Kumar Sandhwar and Prasad, 2019; López-Fonseca et al., 2009). The precipitate could be collected by centrifugation or using a gravity separator. Several approaches are possible for the separation of glucose and, EG. While distillation could be considered due to the different boiling points, the energy requirements would be prohibitive given the high water content. Alternative solutions include membrane separation or enzymatic purification, but these may add complexity and cost to the process (Osman et al., 2024). Another option may be to further ferment the remaining, EG and glucose to obtain high-value chemicals, thus making separation unattractive (Vikram Pandit et al., 2021; Wagner et al., 2023).

## 6 Discussion

Although SSD is a promising strategy for recycling textile waste, the real performance of the spores remains unclear. Firstly, at the cellular level, the different combinations of spores with displayed enzymes need to be addressed. With three cellulases and one PETase, 15 different spores are theoretically possible, considering one, two, three or four enzymes on one spore (see Table 2). Furthermore, considering the 33 different spore proteins suitable for anchoring opens the way to even more possibilities (Todd et al., 2024). Given the combinations of designed spores in the process, the optimal combination also needs to be clarified. Secondly, at the enzyme level, the consequences when multiple enzymes are anchored to the spore surface cannot be foreseen, yet. This uncertainty comes along with the usual problem for immobilized enzyme systems, i.e., for SSD technology, it has not yet been conclusively clarified whether it represents an advantageous trade-off between stability and catalytic activity of the anchored enzymes. The access to the substrate is significantly different for the anchored enzyme compared to the free form, since it moves with the spore. However, if several enzymes are fixed on the spore, several substrate molecules can probably be processed simultaneously while adsorption/desorption kinetics might not remain the same. In terms of stability advantages, they also depend in part on how the process is designed, as increased thermostability is only advantageous if the enzyme must be active at higher temperatures. Thus, the proposed recycling process calls for such a solution whereas a different process design might not. At least, it is known that the SSD enzymes can be used for several reaction cycles without significant loss of the catalytic rate, e.g., for degrading the substrate p-Nitrophenyl butyrate to p-Nitrophenyl a catalytic rate of 84% was reported after the recombinant spores were used for three reaction cycles (Chen et al., 2015). Finally, at the process level, parameters must be experimentally evaluated at the reactor scale to define the optimal operating conditions. This will allow the effects of enzyme-textile interactions, mixing speeds, pH values and temperatures to be determined. Overcoming these hurdles is crucial for scaling up the SSD-based process to an industrial level.

**TABLE 2:** Explanation of the total number of spore-enzyme combinations possible with the four enzymes PETase, Endoglucanase, Exoglucanase and 1,4- β -glucosidase. The conducted combinatorial analysis is based on no repetitions and without respecting the order of the combinations. Thus, only unique combinations of enzymes on the spore are counted.

Enzymes on spore	Combinatorial analysis	Possible combinations
1	41	4
2	42	6
3	43	4
4	44	1
Total Number of Combinations		15

## 7 Conclusion

In this work, current approaches for cotton/PET mixed fibre recycling were reviewed. The SSD Technology applied to *B. subtilis* was presented as a promising strategy for mixed fiber recycling. Due to the stability and reusability of spores, combined with the specificity of immobilized enzymes required for PET and cellulose degradation, it expands the toolbox of recycling technologies to reduce textile waste. Compared to traditional enzyme applications, the ability to immobilize multiple enzymes on a single biological platform is advantageous regarding process efficiency and cost reduction. To facilitate the up-scaling of this technology in case of successful advances in SSD, an appropriate process design has been proposed. This approach contributes to a circular textile industry by returning textile waste back to the production cycle. Future developments should focus on optimizing enzyme combinations and spore display effectiveness.
